# Supplementary material for: Metagenomic estimation of absolute bacterial biomass in the mammalian gut through host-derived read normalization
Source: bioRxiv. 2025 Jan 7:2025.01.07.631807. Preprint. [Version 1] doi: 10.1101/2025.01.07.631807 (PMC11741328; doi:10.1101/2025.01.07.631807)
Supplement: Supplement 1 [file NIHPP2025.01.07.631807v1-supplement-1.pdf]

bioRxiv preprint doi: https://doi.org/10.1101/2025.01.07.631807; this version posted January 8, 2025. The copyright holder for this preprint (which was not certified by peer review) is the author/funder, who has granted bioRxiv a license to display the preprint in perpetuity. It is made available under aCC-BY 4.0 International license.

## Supplementary Information

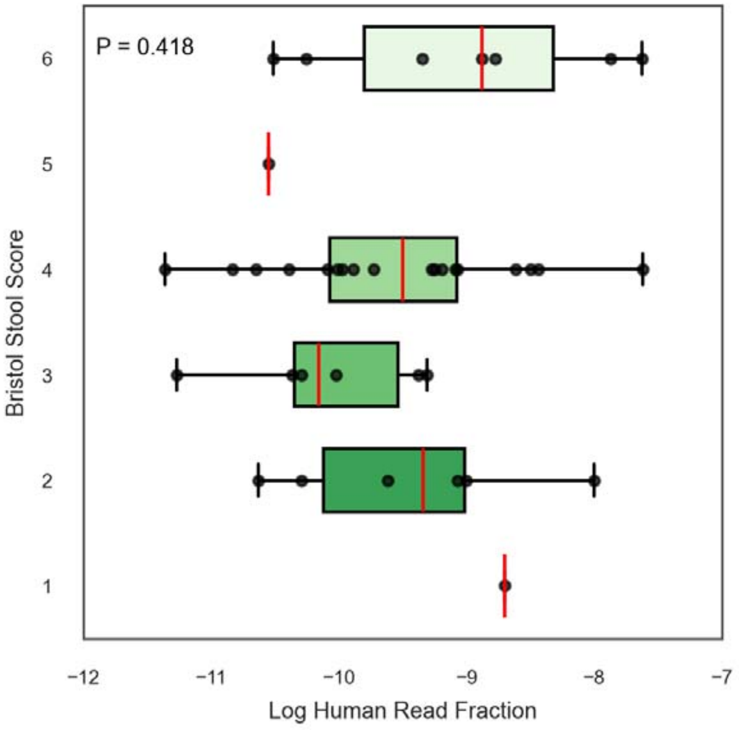


**Figure S1. Distribution of log-transformed human read fractions across different Bristol Stool Scores.** Boxplots showing human read fractions (relative to total metagenic reads) across Bristol stool score categories (n = 39). Each boxplot displays the center line (median), box limits (first and third quartiles), and whiskers (1.5 × interquartile range). Using ordinal logistic regression, we did not observe a significant association between human read fractions and Bristol scores (P=0.418).
